# A subtle economy of time: Social media and the transformation of Indonesia's Islamic preacher economy

**DOI:** 10.1002/sea2.12075

**Published:** 2017-01-11

**Authors:** Martin Slama

**Affiliations:** ^1^ Institute for Social AnthropologyAustrian Academy of Sciences1020 ViennaAustria

**Keywords:** Social Media, Islam, Indonesia, Temporality, Bourdieu

## Abstract

The article is concerned with the latest developments in Indonesia's Islamic field. Its focus is on the role of social media in exchange relationships between Islamic preachers and their constituency. The article first discusses economic exchanges between preachers and their followers, and then it concentrates on social exchanges and how they are mediated today. Empirically, the article delivers insight into the concerns of mostly female Indonesian middle‐class Muslims and shows how preachers have to adjust to the needs of their followers who are regularly online. Theoretically, the article offers a rereading of Pierre Bourdieu's classic work on forms of capital and their conversion. It emphasizes the temporal dimension of capital accumulation and conversion and explores the temporalities of online exchanges that have become constitutive of preacher–follower relationships. In doing so, it shows how Indonesia's Islamic preacher economy is currently transformed by these online exchanges, resulting in preacher–follower relationships that are characterized by dialogic constructions of Islamic authority. Being part of Indonesia's Islamic field, these changes in the Islamic preacher economy point to a broader trend in Indonesia's Islamic field toward greater sensitivity to the needs and worries of Indonesian middle‐class Muslims.

Indonesia's Islamic field has undergone several significant developments in the last decades. Islamic currents that have their origin in other parts of the Islamic world have increasingly been introduced into Indonesia, giving rise to new Islamic organizations, networks, and mobilities (Bowen 2008; Machmudi 2008; Noor 2012; Barendregt 2009). An expanding middle class has discovered Islam for itself, fusing Islamic practice with various forms of consumption (Fealy 2008; Jones 2010; Slama 2014; Rudnyckyj 2015). Sufism, traditionally practiced in rather remote Islamic boarding schools (*pesantren*) under the guidance of a charismatic teacher (*kiayi*), has found its way into Indonesian cities, where today it informs the Islamic practice of white‐collar employees (Zamhari and Day Howell 2012; Day Howell 2015). At the same time, reformist interpretations of Islam have transcended its traditional core constituency of urban trading communities and can be found now among the rural population and lower‐class urbanites (Hefner 2009; Wildan 2013). As in other parts of the Islamic world, mass media have incorporated Islam into their programs, and Muslims use a variety of media for their own purposes in sometimes unique and surprising ways. Especially owing to the media's entanglements with Islam, new Islamic figures have emerged that embody new types of Islamic authority (Watson 2005; Hoesterey 2008, 2016).^1^ Additionally, Islamic ways of handling economic issues have gained popularity, ranging from Islamic banking to Islam‐inflected self‐improvement programs aiming at enhancing piety and productivity (Hefner 1996; Juoro 2008; Sakai 2008; Rudnyckyj 2010).

This article is located amid all these changes by focusing on the latest developments of Indonesia's Islamic field. It is concerned with the uses of social media for religious purposes, having significant ramifications for what I call the Islamic preacher economy. After discussing the economic exchanges this economy comprises, the article is concerned with its symbolic dimensions, especially with linguistic and emotional exchanges that occur on social media. To be able to grasp the entanglements between the economic and symbolic dimensions of Indonesia's Islamic preacher economy, the article resorts to Pierre Bourdieu's (1986) conceptualization of different forms of capital and their conversion. The reading of Bourdieu this article offers has a special emphasis on the temporal aspects of capital accumulation, capital conversion, and the exchanges that are inherent in these processes. Such a reading—or, better, rereading—of Bourdieu allows attention to be directed toward the temporal subtleties that inform Indonesia's Islamic preacher economy and its transformations in today's era of the continuing rise of social media.

In Indonesia, the preacher, or *ustadz* (*ustadzah* is the term for a female preacher),^2^ evolved rather recently into a relatively autonomous Islamic institution. The conjunction of two developments seems to be crucial in this regard: the rise of an urban Islamic middle class that likes to express its religiosity through the organization of so‐called study gatherings, or *majelis taklim,* where Islam is practiced often under the guidance of an *ustadz*. Middle‐class Indonesians arrange such gatherings in homes or in office buildings; they often prefer smaller prayer sessions involving family members, neighbors, friends, and colleagues, yet they also organize meetings in the mosques of their middle‐class neighborhoods or on university campuses. In the meantime, among the lower classes, such gatherings have become popular as well, with people mainly meeting in mosques or following the most prominent preachers to wherever they hold their mass gatherings. These preachers can attract crowds of thousands of people (Millie 2009, 2011; Woodward et al. 2012).^3^

As a consequence, in the last two decades or so, a huge market for Islamic preachers has emerged in Indonesia, which the Islamic scholars from the already established Islamic organizations and centers of learning were only partly able to seize. There was room for new preachers, who sometimes had rather unusual credentials, as their CVs often lacked what representatives of the Islamic establishment would regard as proper Islamic education. This leads to the second main development that has informed today's Islamic preacher economy: Some preachers started to appear on Indonesia's mass media, especially private TV stations, and developed *majelis taklim* programs that resembled the popular Islamic gatherings that had emerged in urban settings.^4^ As a result, some of these preachers, male as well as female, became immensely popular and a kind of role model for the many more locally operating preachers who can be found throughout the Indonesian archipelago (Nisa 2012; Slama 2012; Winn 2012). In Indonesia today, *ustadz* are almost everywhere, as are *majelis taklim*—urban, rural, upper class, lower class, on‐air, off‐air, online, offline, and so on. Many Muslims engage in this particular Islamic sociality where the Qur'an and other Islamic texts are read and, most importantly, where the *ustadz* give sermon and advice.

To speak of a preacher economy here is justified for several reasons: Being an *ustadz* has become something like a profession, including the acceptance of material rewards that allow *ustadz* to make a living from preaching (as will be discussed in the next section in detail). This economy is embedded in a market that determines the value of the preachers, ranging from high‐priced celebrity *ustadz* to less prominent *ustadz* who have not (yet) managed to live on their preaching activities. In addition, the preacher economy not only comprises economic transactions with regard to preaching per se but also fuels broader Islamic consumption patterns from which *ustadz* can gain income. In light of these evident economic aspects, it is necessary to discuss this material transactional side of Indonesia's Islamic preacher economy first. This discussion will provide the basis for a broader analysis of Islamic uses of social media and their temporal dimensions as part of this economy.

## Indonesia's Islamic preacher economy

As applies for the majority of religious activities, on the surface, Indonesia's Islamic preacher economy is not about accumulating economic capital in the first place. It's not about money; it's about practicing one's faith, as many involved in this field assert. Nevertheless, a closer look at *majelis taklim* activities reveals that they are not devoid of economic aspects, if not of quite obvious economic interests. This led one observer of Islam in Indonesia to speak of “markets of faith” (Abaza 2004), suggesting that religious and economic activities often intersect, without suggesting that the former can be reduced to the latter. For example, one can find preachers who run businesses, such as selling Islamic clothing or various products from the Middle East, such as dates in Ramadhan, and who own travel agencies that offer pilgrimage tours. In addition to that, preaching itself can generate a considerable income (see also Hefner 2009:69). In many parts of Indonesia, it is common for preachers to receive money decently handed over in an envelope every time they preach. The amounts these envelopes contain can differ considerably, depending on the class position of the *majelis taklim* members. This applies also to the currency used. As one prominent preacher who regularly appears on television told me, in Jakartan elite circles, the envelope is usually filled with dollars, not with rupiah (Nasarudin Umar, personal interview, July 4, 2014).^5^ And there are preachers with a middle‐class constituency who prefer to have the money transferred directly to their bank accounts. This procedure is also used when a preacher is invited to another city. As prominent *ustadz* like to travel with an entourage, funds for the plane tickets have to be provided in advance (Mrs. Wiwin, personal interview, February 2, 2015).^6^

Stars have their price, and how much they cost is closely related to their media appearances. This is the case with Ustadz Maulana, one of Indonesia's most prominent preachers, who has a permanent contract with Trans TV, a private Indonesian TV station, for his show *Islam Is Beautiful* (*Islam itu Indah*). As one interlocutor told me, those who intend to invite Ustadz Maulana have to prepare funds that can match what he earns from his TV appearances. Such *ustadz* are said to have a *tarif* (rate) for which they can be hired (Mrs. Wiwin, personal interview, February 2, 2015). Because of these high expenditures for celebrity preachers, it is not uncommon for the main host to try to share the costs by asking other institutions, such as private companies, if they want to have the *ustadz* give a sermon at their places as well.^7^

Yet Indonesia's Islamic preacher economy certainly does not only consist of such celebrity preachers. In fact, most *ustadz* operate on the middle and lower rungs of the economic ladder. They receive an amount that equals a handful of dollars for one appearance or, in poor neighborhoods, are offered an allowance in kind, such as fruits and vegetables. In some remote regions of Indonesia, as I could observe in the Moluccas, it can also happen that the *ustadz* does not receive anything for preaching, except for a big thank‐you.^8^

At this point, an analysis that solely focuses on the economy of Indonesia's Islamic preacher economy, that is, on economic capital, reaches its limits. Islamic preaching clearly cannot be reduced to “rational” economic calculation with the aim of generating profit. Yet one can still regard it as an economy if one broadens the term's semantic field and associates “capital” not only with the economic realm per se. Such a step is necessary to be able to analyze not only the current dynamics of Indonesia's Islamic preacher economy but also its transformations in today's social media age. When it comes to multiple notions of capital, obviously Pierre Bourdieu's work becomes relevant. In what follows, I thus revisit Bourdieu's conceptualization of different forms of capital, their conversion from one form into another, and, most important, the temporalities that are constitutive of these processes.

## Bourdieu's forms of capital revisited in Indonesia's Islamic field

In his classic article titled “The Forms of Capital,” Bourdieu (1986), following Marx, generally defines capital as accumulated labor, and he distinguishes three main types of capital, namely, economic, social, and cultural. A crucial observation I adopt from Bourdieu in this regard is that the accumulation of different forms of capital implies different temporalities. For example, it takes much more time to accumulate cultural capital in the form of higher education, let alone through the whole process of socialization into a middle‐ or upper‐class family, with all the values, tastes, and bodily dispositions this implies, than economic capital in a financial system that knows phenomena like “windfall profits.” The forms of capital are strongly interlinked, and the possession of economic capital serves often as the basis for the accumulation of the other types of capital. Economic capital provides the upper classes not only with the money but also with the time to engage in practices that enhance their cultural and social capital by investing time in formal and informal education as well as in “a durable network of more or less institutionalized relationships,” as Bourdieu (1986:248) defines social capital. It is in these upper strata of society where the conversion of forms of capital is most successfully practiced, because being able to spend time on particular cultural and social activities is itself already a conversion of economic capital into cultural and social capital. And the time invested in obtaining cultural and social capital pays off in the long run, when these forms of capital are converted into economic capital—higher payment because of better education, better jobs because of better connections, and so on.

Yet again, as Bourdieu emphasizes, capital conversion comprises different forms of exchange, which, accordingly, rest on different temporalities. He distinguishes between economic and social exchange in particular: “In contrast to the cynical but also economic transparency of economic exchange, in which equivalents change hands in the same instant, the essential ambiguity of social exchange … presupposes a much more subtle economy of time” (Bourdieu 1986:252). Bourdieu obviously alludes to classic anthropological fields of research like gift exchange, a process in which it is crucial that there be a time difference between the gift giving of two groups or persons. In such contexts, immediate reciprocity would be regarded as an insult rather than as a welcome maintenance of relationships. However, when to return a gift or a favor, if not ritualized, can be a tricky question and is, as Bourdieu points out, inherently ambiguous—a subtle and by no means transparent economy of time indeed.

Bourdieu's theory of the forms of capital and their conversion, one has to add, is based on an analysis of French postwar society, especially on its educational system, as well as on his fieldwork in rural Algeria (Bourdieu 1977). These are contexts that differ considerably from current processes in Indonesia's Islamic field with which this article is occupied. However, taking a Bourdieuan perspective on the Islamic preacher economy in Indonesia reveals not only the temporalities of capital conversion in this field but also the temporalities of social exchange and, with them, how profound transformations of the field are occurring. These transformations are, of course, recognized by the preachers themselves, often in the form of critical remarks reflecting the struggles that are currently taking place in their domain.

Not so long ago, an Islamic scholar's reputation depended mainly on his religious education: where, at which institution, with whom, and how long he had studied (and also family members, such as his father, if he came from a prominent family of Islamic scholars). In other words, a particular amount of time, in some cases spanning one's whole youth and early adulthood, had to be invested to accumulate the cultural capital potentially available in Indonesia's Islamic field. However, especially with the rise of electronic media, this began to change, and we can observe a situation in Indonesia today where some of the most prominent preachers cannot show the credentials that the preceding generation needed to occupy a respected position in the field (Watson 2005; Hoesterey 2016). Recently, Ahmad Mustofa Bisri, a prominent representative of Nahdlatul Ulama, Indonesia's largest Islamic organization, usually characterized as “traditionalist,” lamented the inflationary use of the title *ustadz* for people who are “not yet worthy of it” (*belum layak*). People who know only one verse of the Qur'an, he said, are already called *ustadz*. And he added, “If they appear on TV, they are already regarded as *ustadz*. Important is their nice religious dress, even though they behave like thugs” (Tempo.co 2015a).^9^ Ahmad Mustofa Bisri also complained that if one uses an Internet search engine “like Google” (*seperti* Google) when looking for Islamic legal opinions, what emerge first are the views of “dubious people” (*orang‐orang yang tidak jelas*): “They don't know [Islam],” he is quoted as saying, “but they master IT” (see Tempo.co 2015b).

What figures like Ahmad Mustofa Bisri are criticizing is, to put it in a Bourdieuan language, the devaluation of the accumulation of cultural capital over longer periods of time due to short‐term gains in Islamic authority rendered possible by being media‐ and tech‐savvy. In addition to the seemingly easy accumulation of cultural capital today, what bothers these guardians of tradition perhaps even more, although Ahmad Mustofa Bisri did not explicitly refer to it, is the smooth and quick conversion of cultural into economic capital that one can observe among these new preachers. Moreover, this phenomenon bothers younger preachers as well, such as Ustadz Ruly Arta Nugraha, who is based in the central Javanese city of Yogyakarta, a center of higher education in Indonesia where one can also observe a vivid religious life. He certainly belongs among those who have “mastered IT,” because his Islamic nongovernmental organization (NGO) Rumah Hati Jogja (House of Jogja's Heart) raises funds for social projects via Facebook and other social media, and he is anxious to show in these online realms that the NGO manages funds properly. Ustadz Ruly explained:

I distinguish three classes of preachers today: The first one concerns those preachers who are looking for fame, for money; they usually tend to be funny, make parodies, entertain people or however you want to call that, I don't know. But there is little essence in what they say. The second group of preachers really do preach, but their methodology is still the old one. They are conservative so that the people who listen to them become weary and bored of it and will run away. The third group of preachers really do preach as well, but they use IT, they use technology. [Ustadz Ruly Arta Nugraha, personal interview, January 30, 2015]^10^

By referring to preachers who are allegedly thirsty for fame and money, Ustadz Ruly makes explicit the economic dimension of the religious field, a dimension that, according to Bourdieu (1991:25), is typically denied. That the religious field must not appear as if it is determined by greed or gain, and that religious figures must give the impression that they are free of personal economic interest, lies at the heart of its legitimacy (see also Bourdieu 1977). Ustadz Ruly's critique thus attempts to delegitimize certain forms of Islamic proselytization in Indonesia by imputing pure economic interest to certain preachers. Those he delineated as belonging to this class of preachers usually use social media, and thus directly compete with Ustadz Ruly, in contrast to his “old‐fashioned,” “boring” colleagues. Moreover, in Indonesia, preachers have become popular who combine Islam with a Weberian “spirit of capitalism” by asserting that God will reward them not only in the hereafter but also in this world in very concrete, material ways if they engage in particular Islamic practices. Consequently, by displaying their economic success, they also demonstrate their closeness to God. And they inspire their followers to follow them on their path to prosperity through piety (Rudnyckyj 2010; Hoesterey 2016). In addition to holding at arm's length the “funny,” entertaining preachers, Ustadz Ruly, who likes to emphasize his modest lifestyle, also distances himself from these wealthy self‐help preachers who somehow manage to fuse cultural and economic capital by making them indistinguishable, as one form of capital serves as proof for the other, and vice versa.

Emphasizing or even showing off wealth and economic success is not only contested within Indonesia's Islamic field but also challenges Bourdieu's overall characterization of the religious field. Identifying piety with modesty (in a material sense) is certainly a value in Indonesian religious circles, but it is far from being hegemonic, as the rise of rich preachers preaching how to become rich shows.^11^ Ustadz Ruly's critique of the popular and rich preachers thus reflects his position in Indonesia's Islamic field—and as the next section discusses in detail, which position a preacher occupies is crucial to how he encounters the larger transformations the field is undergoing in connection with the introduction of social media.

## Social media, social capital, and a subtle economy of time

Capital accumulation and conversion certainly work best for the stars among the preachers who have TV contracts and excellent connections to Indonesia's economic and political elites. Yet for the large majority of preachers, who operate on a more local level, things have become more complicated, especially since social media are part of the preacher economy. Today the participants of study gatherings, or *majelis taklim,* as well as the preachers are increasingly going online. In middle‐ and upper‐class circles in particular, the members of these groups are often connected via Facebook and by the instant messaging services such as BlackBerry Messenger (BBM) or other communication programs like WhatsApp, LINE, and Telegram. These applications allow for the formation of groups in which preachers are also involved. This means that group members can easily consult their preacher, raising issues from Islamic jurisprudence to personal problems. Through these particular online mediations, the Islamic prayer group that, for example, meets only once a week offline becomes part of everyday life, as does the preacher, to whom messages can be sent anytime.

The online availability of preachers can reach a level of necessity that, if a preacher does not yet own a smartphone or a similar device, it is simply given to him by his middle‐ or upper‐class followers. This happened to Ustadz Hasan, a teacher of Islamic religion in a high school in South Jakarta who runs several *majelis taklim* and was equipped with an iPad by his followers. He also works as a private religious teacher and became popular among Indonesian celebrities. When I met him, he presented a picture to me on his iPad that depicts him together with Indonesian pop star Maya Estianti. However, Ustadz Hasan preaches among people from all walks of life, as some of his *majelis taklim* are meant for lower‐class urbanites. Based on his experience, Ustadz Hasan likes to distinguish his followers according to their class background, asserting that “with middle‐ to upper‐class people communication is usually two way” (Ustadz Hasanuddin, personal interview, July 10, 2014).^12^

This two‐way communication facilitated by social media is also used by his followers to engage in private conversations with Ustadz Hasan. He explained that his followers ask for his advice concerning “family problems” (*masalah keluarga*) such as affairs or divorce (Ustadz Hasanuddin, personal interview, July 10, 2014). In such cases, communication indeed runs two way, relying on—and sometimes challenging—the authority of the *ustadz* at the same time. In other words, social media affect the preacher economy in a way that Islamic authority is now constructed more dialogically than before. Preaching today consists not only of talking to a religious crowd, whether directly or via electronic media, but also of personal exchanges between the preacher and his followers. Of course, this is not a completely new phenomenon, as *majelis taklim* gatherings sometimes also comprise a Q&A session, and people sometimes like to visit the *ustadz* at home, if they have a serious issue to discuss. But this “two‐way communication,” as Ustadz Hasan put it, has now multiplied and it is much easier to accomplish, precisely because it occurs online, where people can avoid emotional dispositions, such as feelings of shame (*malu*), that are heavily invested with meaning in Indonesia (Boellstorff and Lindquist 2004; Slama 2010, 2016; Barendregt 2012).

Taking the perspective of preachers, and coming back to Bourdieu's theory of forms of capital, for them, social media provide new opportunities to accumulate social capital, as preachers can maintain their relationships with well‐to‐do, media‐savvy middle‐class Muslims more easily, which allows them to convert social into economic capital more easily as well. Yet there is another aspect of this phenomenon that deserves closer attention. It is closely related to the temporal dimension of social exchange. The following ethnographic examples illustrate this crucial point.

WhatsApp user Mrs. Wiwin from Yogyakarta is in regular contact with a preacher whose name is Ustadz Eko. Unfortunately, a few years ago, her oldest child died, and sometimes she has difficulties coping with this tragic loss. She then writes Ustadz Eko a message, and usually she does not have to wait long for a reply. She also told me of another *ustadz* who has already reached a kind of celebrity status and who likes to reply the next day, if at all. But on the next day, Mrs. Wiwin explained to me, her emotional state has already changed. She also said that she actually knows what to do in such situations but is nevertheless looking for “the support” of the *ustadz,* a support that should come in due time (Mrs. Wiwin, personal interview, February 2, 2015).

Urgency is an important aspect of “the subtle economy of time” that characterizes exchanges on social media. Yet even in urgency, there is subtlety, because preachers have to learn how to deal with their followers' demands for online communication. Ustadz Eko knows Mrs. Wiwin and her problems very well. They regularly meet at his *majelis taklim* gatherings, and their online exchanges have become part of their daily lives. Ustadz Eko is thus aware of the emotional dimension of their exchanges that necessitates a particular temporality. One can thus easily imagine that for some preachers, online communication can become not only a convenient way to spread a message but also quite challenging. These kinds of online encounters cost time and involve emotional support. They are part of a broader set of phenomena that James Hoesterey (2016:60) has called “affective exchanges” in his analysis of the rise and fall of popular TV preacher Aa Gym. However, whereas older media like television have a clear temporal structure and allow only for a limited kind of affective exchange (being complemented by emotionally choreographed offline meetings with his fans in the case of Aa Gym), online forms of two‐way communication can become in temporal as well as emotional terms much more intense. From the perspective of the preacher, his new job as online consultant is therefore not always so easily integrated into daily offline life, as the following example shows.

In August 2014, I met Ustadz Lubis—whom I had known for many years—in a shopping mall in the south of Jakarta.^13^ Because Ustadz Lubis is aware of my research interests, it wasn't long until he referred to a message he had received on the same day from a woman who lives in Bekasi, an eastern suburb of Jakarta. The woman was advanced in pregnancy and complained about her husband, who accepted work in central Jakarta and thus was not able to care about her to an extent she would have found appropriate.^14^ In contacting Ustadz Lubis on BBM, she hoped that the preacher could convince her husband to quit his job in the city center and return to Bekasi. When Ustadz Lubis had finished reading the message aloud to me, he sighed deeply and said that time and again he has to meddle (*campur tangan*) in the households (*rumah tangga*) of other people. “But it wouldn't be nice not to answer,” he also said. So he formulated his reply in my presence, basically arguing that the woman should be happy to have a husband who knows his duty to provide for the family. Only if the family income were already secured could the woman ask him to quit his job (Ustadz Lubis, personal interview, August 9, 2014). In 2014, Ustadz Lubis had 74 BBM contacts, and not all messages he received, on some days up to 20 or more, were related to private affairs. They ranged from simple questions about Islamic practice, such as the right way to wash certain parts of the body before prayer, to more complicated cases, such as the preceding. Accordingly, he answered some questions on the following day or collected questions and answered them later all at once, sending his advice to all participants of the BBM groups he is heading.

In a similar way, social media are used by Ustadz Nabil Assegaf, a preacher also based in Jakarta, who runs—in addition to his offline *majelis taklim*—groups on WhatsApp and Telegram. He has a very positive attitude toward social media. Yet he also told me that his wife likes to remind him when he spends too much time with his smartphone or tablet replying to followers' messages. When I asked him about the communication that takes place between him and his followers, he explained,

At the *majelis,* but mostly via this one [pointing at his smartphone]. Every day my work is answering questions. This problem and that problem, problems between husband and wife, problems with naughty children, various things, a lot! And also religious questions, thank God, this device makes it much easier. [Ustadz Nabil Assegaf, personal interview, July 14, 2014]

Although Ustadz Nabil embraces social media as part of his preaching activities, his account nevertheless comprises ambivalence. On one hand, he appreciates the ease with which he can reach his followers; on the other, the same ease of access allows his followers to reach him and to convey a seemingly infinite series of problems. Giving advice to solve these problems has become his “work” (*pekerjaan*).

These examples point to the subtle economy of time on which Indonesia's Islamic preacher economy is based today. There are no clear rules for the temporalities involved in these social exchanges, but the preachers who want to enter this market have to develop a sense of how much time can pass until they reply to particular messages without disappointing their followers. When Mrs. Wiwin writes a message about the loss of her child or the woman from Bekasi about her husband working in central Jakarta or another person about the proper way to wash one's body before prayer, this implies different expectations of how long they have to wait for a reply. Developing a sense of when to write back is important for the preachers, because with these relations of online exchange, their followers enjoy what James Hoesterey (2016:19) has called in the context of TV preachers “a new form of consumer power” (2016:19), and preachers who do not develop the skill of finding the right words at the right time are easily abandoned.

Compared to other forms of verbal exchange and encounter between preachers and their followers, social media put the preachers in a more vulnerable position. At the same time, their followers enjoy the quicker communication and a high degree of privacy and intimacy. Meeting an Islamic authority at home often means having to wait until the preacher is ready to receive his guest or until he has finished conversations with other guests who had arrived earlier. Sending letters to magazines, which mainly employ psychologists but also religious experts, also involves a long wait time until the letter is chosen by the editors. Radio call‐in shows, another older media format that is available in Indonesia to raise personal issues, might not be considered as an option in many cases because of the rather low level of privacy. On social media, however, a mix of speed and privacy produces the intimate and affective relationships that many middle‐class Muslim women are seeking. Quick replies provide a sense of copresence, that is, that one shares not only the same virtual space but also a temporal realm with the preacher.^15^

Yet not only preachers have to develop a sense of time and timeliness to maintain relationships. To minimize the time lag of online exchanges, some followers closely watch the social media habits of their favorite preachers. Mrs. Rita, for example, a mother of two children from Yogyakarta whom her husband recently divorced, likes to participate in various *majelis taklim* and thus knows a lot of preachers. Mrs. Rita likes to send messages to prominent preachers from whom it is usually rather difficult to get a personal answer. Given her difficult situation after her divorce, she appreciates it greatly if she doesn't have to wait too long for a reply. Similar to Mrs. Wiwin, she is looking for emotional support. To attract the attention of her favorite preacher, she developed the following strategy:

Usually I wait for a long time. But sometimes, rather than waiting, I like to raise topics that are dear to my heart early in the morning. I noted that the *ustadz* usually sends out his messages at the time of the morning prayer. When my smart phone makes “ting” early in the morning, it is surely the *ustadz*. Then I directly approach him with my problems. And he answers! Communication can be quick, if we do it like that. [Mrs. Rita, personal interview, August 6, 2014]^16^

Mrs. Rita's account exemplifies, so to say, the other side of the coin of the subtle economy of time with which we are dealing here. The time of a prominent preacher is a particularly scarce resource, and Mrs. Rita discovered a time slot in the morning when she can get access to this resource. In this reverse case, rather than preachers having to determine when to answer their followers, the followers try to detect when the preachers will reply. The question of time is obviously a question of power in Indonesia's Islamic preacher economy and serves as a major indicator of whether a preacher's popularity, that is, the might of his celebrity status (if he enjoys such a status), can prevail over the consumer power of his followers, or vice versa.

A preacher to whom Mrs. Rita and the aforementioned Mrs. Wiwin referred when it came to belated replies was Ustadz Wijayanto, who commutes between Yogyakarta, his hometown where he still regularly appears in *majelis taklim,* and Jakarta, where he stars in religious TV shows. When I met Ustadz Wijayanto at his home in Yogyakarta, he told me that every day he receives at least 100 messages, and on that particular day of our encounter, he had received around 300, adding that he does not reply to all and that he likes to delay his advice. Before he goes to sleep at night, he replies only to those messages that he deems “necessary.” But what falls under this category can vary. Ustadz Wijayanto showed me a message from a woman who asked him why women usually do not join their husbands in the mosque for the morning prayer. Instead, they pray alone at home, which she finds regrettable. He then explained:

I consider the priority. This example is actually not that urgent, but since I know the woman well—she once was my student at Gadjah Mada University^17^—I give precedence to her. Of course also in cases of urgency I prioritize messages, for example if people struggle with *broken heart* or *broken home* [English in original]… . But what concerns legal questions—Am I allowed to do this or not?—I usually choose to postpone the answer. Because I'm realistic, every day I'm *teaching, training, shooting* [English in original]. Almost every day I'm occupied with shootings at the TV station. [Ustadz Wijayanto, personal interview, March 2, 2016]

Ustadz Wijayanto can afford to prioritize certain messages or to ignore others. Yet he does not arbitrarily do so. He devotes more of his time to the personal problems (“broken heart, broken home”) of his followers than to the Islamic legal issues that they bring up. And followers he knows for many years, for example, from the time when he was not yet famous, can also expect to have their questions answered soon. The temporal hierarchy that is inherent in Ustadz Wijayanto's social media practice points to the larger subtle economy of time on which Indonesia's Islamic preacher economy is based, where emotional intimacy finds it expression in the temporal subtleties of online exchange. It is thus not a coincidence that Ustadz Wijayanto chooses to further invest in longtime relationships as well as in cases of emotional urgency. By doing this, he retains a practice—and to a certain extent also an image—that is associated with the majority of preachers who have not reached celebrity status and who personally care about their followers, while at the same time he necessarily disappoints followers who receive less or no attention, such as Mrs. Rita and Mrs. Wiwin. In fact, popular *ustadz* like Ustadz Wijayanto no longer depend on the social capital preachers can accumulate by maintaining personal relations with their followers via social media, although they might still engage with a limited number of them online. They operate in a realm of less personal communication channels, that is, in the realm of mass media, that generate a mass following, which can also be managed by social media like Facebook and Twitter, but not by running small WhatsApp and BBM groups, as many less prominent preachers do.^18^

## By way of conclusion: (Re)reading Bourdieu and his critics

My rereading of Bourdieu with a special focus on the temporal aspects of his theory of capital accumulation and conversion allows me to conclude that in Indonesia's Islamic preacher economy today, the conversion of cultural into economic capital, that is, the conversion of one's status as a preacher into an income, increasingly depends on how preachers can accumulate social capital through the use of social media. The labor that is necessary for doing this consists not only of the accumulated labor of the past, that is, the time the preacher has invested in studying Islam and in learning to preach, but also of the labor he is able to invest in the here and now to uphold relations with his followers. The temporality of symbolic taking and giving, that is, the time difference between receiving a message and replying to it, can be crucial to gaining and upholding status. A preacher who copes well with the subtle economy of time of online exchanges can accelerate the conversion of his social and cultural capital into economic capital. These changes in the preacher economy explain the critical remarks of older prominent Islamic leaders about preachers who “master IT.”

The point this Bourdieuan analysis allows me to make is that the transformation Indonesia's Islamic preacher economy is currently undergoing rests not only on a pluralization of religious authority, though this is an important aspect, but also on a new principle of how religious authority is constructed, namely, on exchanges that demand particular temporalities—depending on the topic discussed and the emotions involved. Preachers who cannot meet the new demands of online exchanges risk losing their middle‐class followers and, with them, large parts of their income. Only the stars among the preachers, those regularly appearing on television and holding mass gatherings where they have become inaccessible for personal relationships, are no longer subject to this logic of exchange, which represents the logic of accumulation of social capital in today's social media age. But this does not mean, as this article also shows, that they have completely distanced themselves from personal online communication.

At first glance, the reader might be surprised that this article relies on the work of Bourdieu to analyze these transformative developments in Indonesia's Islamic field. The critique perhaps most frequently put forward against Bourdieu is that his concepts are difficult to apply in analyzing social change and that he puts too much emphasis on the dominating structures of society, ascribing too little room for subversive thought and action. In short, his work lacks concepts for examining phenomena like agency and resistance. As Hugh Urban (2003:362) has aptly summarized, another major critique concerns his use of economic metaphors to explain the social world or a particular social universe, such as the religious field, where specialists like prophets, priests, or, in our case, Islamic preachers “struggle over material and symbolic resources.” Very much in line with the critique he sums up, Urban (2003:367) does not mince his words when he presents his own view of Bourdieu: “In a sense, Bourdieu's model is even more problematic than Marx's, because Bourdieu has turned us all not simply into laborers, but into *capitalists,* self‐interested beings who seek to accumulate and maximize our own symbolic and economic capital.” And he adds that if one follows Bourdieu, “*all action at every level becomes capitalist*” (Urban 2003:367). Instead, Urban (2003:384) argues that “religion does not only play a reproductive role in legitimating the dominant status quo in a given socio‐political formation,” and it is precisely in the realm of religion where “human beings are not always or solely governed by the pursuits of self‐interest or acquisition of capital.”

The reading of Bourdieu that I present in this article clearly differs from this mainstream critique. One could even say that if this critique were correct, it would have been impossible to analyze something like Indonesia's Islamic preacher economy, a field that currently embodies transformation and change, by relying on Bourdieu. Similarly, defining Indonesian Islamic preachers as capitalists “at every level” would deny us the ability to analyze the subtle forms of exchange—economic and symbolic, offline and online—that are so crucial to how the relations between preachers and their followers are established and maintained. Using Bourdieu's economic metaphors to understand Indonesia's Islamic preacher economy does not automatically turn all actors in Indonesia's Islamic field into rationally calculating individuals in search of material and symbolic gain. The question whether Islamic preachers preach to become wealthy and celebrated, as some of them are indeed accused of, is missing the point. For whatever reason preachers enter the preacher economy, ranging from “pure” intentions of spreading the word of Islam and simply making a living to strategies of becoming famous and rich, today they have to meet the challenges introduced by the subtleties of online exchange. My rereading of Bourdieu's theory of forms of capital and their conversion thus emphasizes the temporal aspects involved. Temporalities are crucial for the processes of capital accumulation and conversion, and they are highly relevant for the social exchanges that are part of these processes. Unfortunately, Bourdieu's theorization of time that, in my reading, lies at the heart of his theory of forms of capital and their conversion remains largely unrecognized by critics like Urban. Yet this does not mean that a Bourdieuan analysis is fully applicable in each and every aspect to what this article is concerned with. In the context of Indonesia's Islamic preacher economy, the limits of a Bourdieuan approach do not lie in his theory of forms of capital (and their conversion) but in his conceptualization of the religious field as a realm where economic motives of actors or the economic consequences of their practice have to be concealed. In contemporary Indonesia, instead, where (social) media play a decisive role in the processes of capital conversion, this denial of religion's economic dimensions is not necessarily the case, and their nondenial sometimes becomes a subject of debate.

As I have tried to show in this article, social media added new possibilities and temporalities of social exchange to Indonesia's Islamic field. The subtle economy of time, being characteristic of these exchanges, particularly affects Indonesia's Islamic preacher economy, which was already subject to different temporalities of capital accumulation and conversion before the rise of social media. In other words, social media introduced an additional temporal dimension, with time having become unusually important in the daily exchanges between the preachers and their (mainly) middle‐class followers. Whereas famous preachers who appear on electronic media like television can easily convert their cultural capital into economic capital, the majority of less prominent preachers are busy with the subtlety of exchanges on social media that boost the preachers' social and cultural capital and eventually also allow the conversion of these forms of capital into economic capital. Whatever material interests the preachers might pursue or not pursue, the temporality of capital conversion, that is, how much time it takes to convert their preaching into an income, depends not only on their Islamic and psychological competence but also on mastering the subtle economy of time that governs online exchanges. To be clear, those preachers who remain offline, or who are not able to develop a sense of the temporal and emotional aspects involved, are the least equipped to obtain and maintain a position in this transforming field. Therefore, for many preachers operating in middle‐class circles, these online exchanges have attained new significance, as their acquired ability to preach offline, that is, their basic cultural capital, and their offline accumulation of social capital seem no longer sufficient to uphold status and income. In light of these developments, one can thus speak of a twofold temporal transformation of Indonesia's Islamic preacher economy, where temporalities of capital conversion and social exchange intersect. These dynamics are part of a greater transformation of Indonesia's Islamic field toward a more dialogic construction of Islamic authority and a preacher–follower relationship that is more sensitive to the needs and worries of (mainly female) Indonesian middle‐class Muslims.

## Notes

1 For the influence of electronic media and particularly the Internet on religious authority in general, see Barker (2005) and Hope Cheong (2013); for the Islamic context, see Eikelman and Anderson's (1999) influential volume and especially Anderson (1999, 2005); and for the “cyber‐Islamic environments” in which authority is constructed, see Campbell (2010:30–35).

2 Although there are prominent female TV preachers in Indonesia, especially on the local level, male preachers usually outnumber female preachers by far. This article has its focus on male preachers and their mainly female middle‐class constituency. Following Indonesian conventions, I use the singular forms of the words *ustadz* (male preacher) and *ustadzah* (female preacher) for the plural as well. I also follow the Indonesian spelling of these Arabic terms (which are derived from Persian). They have already become part of the Indonesian language and are used according to Indonesian grammar, which does not differentiate between singular and plural forms of nouns.

3 In addition to *majelis taklim,* other gatherings, or *majelis,* became popular, such as *majelis zikir* (where *zikir* litanies are recited) and *majelis salawat* (where the Prophet Muhammad is praised in prayers and songs) among Indonesian Muslims who like to join Sufi rituals (Zamhari and Day Howell 2012).

4 For the study of Christian uses of electronic media in the context of this rise of Islamic programs in Indonesia, see Barkin (2013).

5 Nasarudin Umar was then deputy minister of religious affairs. The figure of Nasarudin Umar, who is a professor at Jakarta's State Islamic University and rector of a private Islamic higher education institution, the Institut PTIQ Jakarta, attests to the plurality of Indonesia's Islamic preacher economy, ranging from academics to preachers with no formal Islamic education at all. I am grateful to Faried Saenong, who arranged the meeting with Nasarudin Umar.

6 Mrs. Wiwin is an active *majelis taklim* member in Yogyakarta who is frequently involved in organizing events featuring *ustadz* who come from other Indonesian cities. The interview was conducted together with Fatimah Husein from the State Islamic University in Yogyakarta. Fatimah Husein is part of the research team of the project “Islamic (Inter)Faces of the Internet: Emerging Socialities and Forms of Piety in Indonesia,” which I am heading (see the acknowledgments). A note on anonymization is necessary here: Preachers who appear in public are not anonymized, whereas the followers of the preachers are. Therefore “Mrs. Wiwin” is not my interlocutor's real name.

7 To some extent, this practice of rewarding preachers with funds can be compared with what Soares (2005) calls the prayer economy in Mali, where Sufi saints regularly receive gifts from their followers. However, a major difference concerns the mobilities involved with the Malian saints living in their hometowns receiving their followers there, whereas in Indonesia today, it is expected that the preachers be mobile. For different Indonesian examples of the connection between mobility and piety, see Silvey's (2007) research on Indonesian domestic workers in Saudi Arabia as well as my analysis (Slama 2011) of networks of Islamic scholars in eastern Indonesia and my (Slama 2014) study of the latest developments of Islamic saint veneration.

8 As Winn (2012) observed for Ambon, *majelis taklim* gatherings usually take place there without inviting an *ustadz*. The *ustadz* I met on Ambon during my fieldwork in early 2015 mainly preach in mosques and cannot make a living from their religious activities alone.

9 This comment about “thugs” (*preman*) probably refers to the Islamic Defenders Front (Front Pembela Islam), an organization that gained a lot of media attention due to the violent acts they regularly commit and justify by asserting that they intend to combat “vice.” The members of this organization always appear in religious dress, as does their leader, a prominent preacher of Hadhrami‐Arab descent (Bamualim 2011; Petru 2015). All translations, of media sources and interviews, are by the author.

10 The interview was conducted together with Fatimah Husein.

11 Let me briefly add here that these discourses are embedded in a cultural context in Indonesia, and especially in Java, where, according to Benedict Anderson's 1990[1972] classic work, the excessive acquisition of wealth is seen as lack of self‐control and thus as spiritual weakness, which is, as Suzanne Brenner (1998) has demonstrated, part of a gender ideology that devalues the economic activities of female traders.

12 I am grateful to Faried Saenong, who arranged the meeting with Ustadz Hasan, as he likes to be called colloquially.

13 Shopping malls are the most popular places where people meet in Jakarta.

14 One has to explain here that especially during the morning and evening rush hours, commuting between Bekasi and the city center can take several hours.

15 In consideration of the new, more vulnerable position of many preachers, Indonesia's Islamic preacher economy cannot be reduced to the classic model of patron–client relationships that were, for example, analyzed by Scott (1976) in his influential book *The Moral Economy of the Peasant*. The preachers are not in the position of patrons; rather, they have to compete for followers in a market that is governed by the subtle economy of time of online exchanges. In cases of particularly rich followers, it is rather the preacher who can become a client. But on the whole, the social relations of the Islamic preacher economy are more ambiguous and flexible—depending on the current position of the preacher in Indonesia's Islamic field (his popularity or celebrity status) and the class position of his followers—than patron–client bonds, with their fixed social hierarchies.

16 The interview was conducted together with Fatimah Husein.

17 Ustadz Wijayanto was once a lecturer at Yogyakarta's Gadjah Mada University. He now runs an Islamic boarding school in Yogyakarta.

18 Without being able to go more deeply into this due to limited space, I just want to briefly add here that whether social media accounts like Facebook and Twitter are managed by the prominent preachers themselves or by their assistants (which is mainly the case), the exchanges taking place there can also spur temporal dynamics beyond the control of the holders of these accounts.
